# Clinical implications of natalizumab Fab-arm exchange in patients with multiple sclerosis

**DOI:** 10.3389/fimmu.2026.1796273

**Published:** 2026-05-08

**Authors:** Liza M.Y. Gelissen, Stefan P.H. van den Berg, Alyssa A. Toorop, Martijn T. Wijburg, Emma C. Tallantyre, Ninotska I.L. Derksen, Floris C. Loeff, Joep Killestein, Theo Rispens, Zoé L.E. van Kempen, the NEXT-MS study group

**Affiliations:** 1 MS Center Amsterdam, Neurology, Vrije Universiteit Amsterdam, Amsterdam Neuroscience, Amsterdam University Medical Centers, Amsterdam, Netherlands; 2 Department of Immunopathology, Sanquin Research Amsterdam, Amsterdam, Netherlands; 3 Department of Rehabilitation Medicine, MS Center Amsterdam, Amsterdam UMC, Vrije Universiteit, Amsterdam, Netherlands; 4 Division of Psychological Medicine and Clinical Neuroscience, Cardiff University, Cardiff, United Kingdom; 5 Department of Neurology, University Hospital of Wales, Cardiff, United Kingdom; 6 Biologics Laboratory, Sanquin Diagnostiek B.V., Amsterdam, Netherlands; 7 Amsterdam UMC, location Vrije Universiteit Amsterdam, Molecular Cell Biology and Immunology, Amsterdam, Netherlands; 8 Amsterdam Institute for Immunology and Infectious Diseases, Immunology, Amsterdam, Netherlands

**Keywords:** extended interval dosing, Fab-arm exchange, multiple sclerosis, natalizumab, PML - progressive multifocal leukoencephalopathy, wearing-off phenomenon

## Abstract

Natalizumab, a monoclonal antibody used to treat multiple sclerosis, can undergo Fab-arm exchange (FAE) with endogenous IgG4, resulting in a monovalent form with reduced avidity and presumed lower potency than the parental bivalent form. Levels of bivalent and monovalent natalizumab depend on the ratio of endogenous IgG4 to total natalizumab, which varies substantially between individuals. The clinical relevance of natalizumab FAE remains unknown. This study investigated the potential clinical implications of bivalent and monovalent natalizumab levels regarding progressive multifocal leukoencephalopathy (PML), extended interval dosing (EID) and wearing-off symptoms, using multiple cohorts of natalizumab-treated patients. Seven natalizumab-associated PML cases were each matched to two controls. Contrary to the hypothesis that higher bivalent levels might increase PML risk, cases showed a trend toward lower bivalent natalizumab levels, although definite conclusions were limited by small sample size. In patients receiving EID, both total and bivalent natalizumab levels decreased compared to standard interval dosing, with a greater median individual decrease in bivalent natalizumab than in total natalizumab (94% versus 78%). Notably, in more than half of patients receiving EID, bivalent natalizumab levels fell below the quantification limit (<0.1 µg/mL). Finally, patients experiencing wearing-off symptoms during natalizumab treatment had lower bivalent natalizumab levels, although this warrants validation in larger cohorts.

## 1 Introduction

Natalizumab, a humanized immunoglobulin G4 (IgG4) antibody, is used as treatment for patients with relapsing-remitting multiple sclerosis (MS). It binds to the α4-subunit of the α4β1 integrin, also known as the very late antigen-4 receptor, on lymphocytes (1). The α4β1 integrin has an important role in the adhesion and transmigration of activated immune cells from blood into the central nervous system (CNS) by interacting with the vascular cell adhesion molecule-1 (VCAM-1) expressed on endothelial cells (2). Natalizumab exerts its therapeutic effect by preventing this interaction, thereby blocking immune cell migration into the CNS (2).

While natalizumab is a safe treatment that rarely causes severe side effects, it increases the risk of progressive multifocal leukoencephalopathy (PML) in patients positive for the John Cunningham virus (JCV) (3). PML is a potentially fatal opportunistic infection of the CNS, caused by reactivation of JCV in patients with suppressed cell-mediated immunity (4). Therefore, JCV status is routinely monitored in patients treated with natalizumab by measuring anti-JCV antibodies in serum. This measurement provides a definite result, positive or negative, as well as an index value, reflecting the level of antibodies against JCV. A positive test result, a high index value, longer treatment duration and the use of immunosuppressants prior to natalizumab are associated with an increased risk of PML (5). Another disadvantage of natalizumab therapy is the occurrence of wearing-off symptoms (WoS), reported in 40-70% of patients (6–10). These are MS-related symptoms, such as fatigue, motor weakness, or sensory disturbances, that worsen toward the end of the dosing interval and typically resolve after the subsequent dose (7).

The standard dosing regimen of natalizumab is 300 mg every four weeks, which can be administered either intravenously or subcutaneously. The minimum effective total serum concentration is estimated to be approximately 1 µg/mL, below which α4β1 integrin desaturation may occur, potentially reducing treatment efficacy (11). However, studies have shown that natalizumab concentrations at the end of standard four-week treatment intervals are substantially higher in most patients than this threshold (12–14). Consequently, extended interval dosing (EID) has been increasingly conducted in recent years (15–17). While maintaining therapeutic efficacy, it was found that EID is associated with a reduced risk of PML (18). It is hypothesized that this effect arises from the lower natalizumab concentrations at the end of each treatment interval (i.e., at trough) achieved by EID, which allow modest lymphocyte migration into the CNS, thereby preserving immune surveillance and preventing JCV reactivation (18, 19).

**Figure 1:**
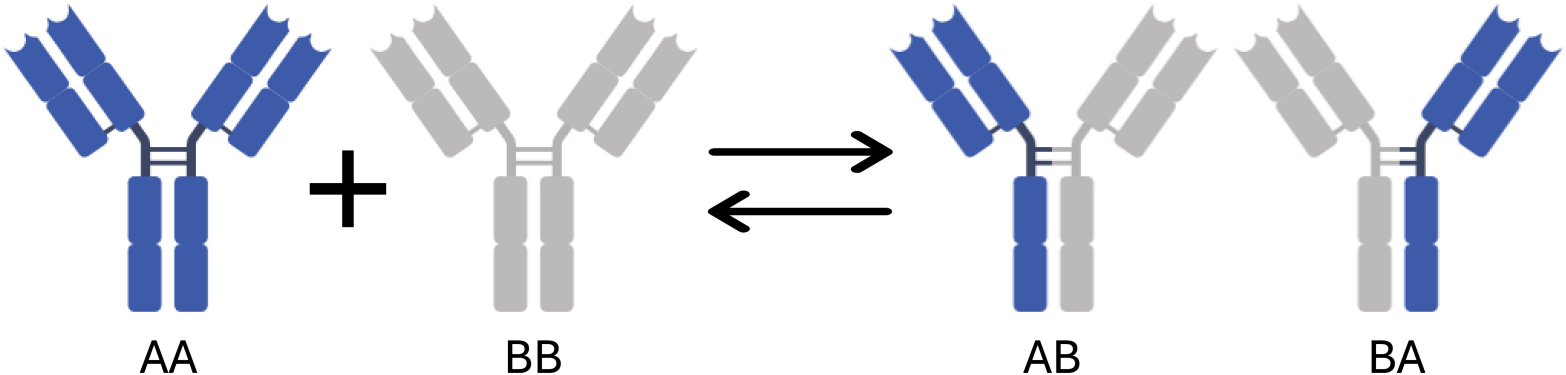
Fab-arm exchange. AA represents a bivalent natalizumab, which is monospecific. BB represents an endogenous IgG4. FAE of bivalent natalizumab with endogenous IgG4 results in monovalent natalizumab antibodies (AB, and BA), which are bispecific.

IgG molecules are generally symmetrical, Y-shaped molecules, with two Fab arms that contain one antigen binding site each, i.e., the molecule is bivalent. However, as an IgG4, natalizumab has the unique ability to exchange half-molecules with endogenous IgG4 in serum (**Figure 1**) (20–22). Because this results in one Fab-arm in the IgG molecule originating from another IgG4 molecule, this process is termed Fab-arm exchange (FAE). Consequently, two forms of natalizumab will be present in serum: an effectively monovalent form and a bivalent form. The monovalent form is bispecific, consisting of one natalizumab arm and one randomly paired, unrelated endogenous IgG4 arm. It was observed that bivalent natalizumab has a 20-fold increased avidity for binding to T lymphocytes compared to monovalent natalizumab, which could result in enhanced potency of the former (23, 24). Studies have shown that the degree of FAE depends on the ratio of endogenous IgG4 to the total natalizumab concentration in serum, leading to different monovalent and bivalent natalizumab levels between individuals, while having similar total natalizumab concentrations (25, 26). This may explain why patients with similar total natalizumab concentrations may exhibit different levels of α4β1 integrin receptor occupancy (27).

So far, it remains unknown whether natalizumab FAE has clinical implications in patients with MS. Therefore, the aim of this research was to explore potential clinical consequences of the variable levels of bivalent and monovalent natalizumab in patients with MS treated with natalizumab. We examined the quantitative relationship between FAE and total natalizumab and endogenous IgG4 levels. We investigated the relationship between bivalent natalizumab levels and PML, with the hypothesis that higher bivalent natalizumab levels permit less lymphocyte migration across the blood-brain barrier, reduce CNS immune surveillance, and thereby increase the risk of JCV reactivation (21, 25, 26, 28). Second, we assessed the effect of EID on bivalent natalizumab levels. Third, we examined whether FAE contributes to WoS, with the hypothesis that lower bivalent natalizumab levels at the end of the dosing interval allow increased lymphocyte migration into the CNS, potentially causing WoS (6, 7).

## 2 Methods

### 2.1 Assays

To quantify bivalent natalizumab in serum, an enzyme-linked immunosorbent assay (ELISA) was developed, using monoclonal antibody anti-natalizumab 2.2 as capture and detection reagent (29). MaxiSorp ELISA plates were coated overnight at room temperature with 0.5 μg/mL anti-natalizumab in phosphate-buffered saline (PBS). The plates were washed five times with PBS/0.02% Tween (PT), then incubated for one hour with patient serum that had been serially diluted (200-25, 000-fold) in high-performance ELISA (HPE) buffer (Essange Reagents), supplemented with 1 mg/mL IMIG (Gammaquin, Sanquin). After washing five times with PT, plates were incubated for 1 hour with biotinylated anti-natalizumab (100 ng/mL in HPE buffer). After washing, 1:10, 000 streptavidin–poly-horseradish peroxidase (poly-HRP; Essange Reagents, Amsterdam, The Netherlands) was added and incubated for 30 minutes at room temperature. After washing, the ELISA was developed with 50% 1-Step Ultra TMB ELISA, Ref 34029, ThermoScientific. The reaction was stopped with 0.2 M H_2_SO_4_. Absorption was determined as ΔOD at 450 and 540 nm. Levels of bivalent natalizumab were quantified based on a serially diluted bivalent natalizumab calibrator. Bivalent natalizumab levels below 0.1 µg/mL could not be quantified and were therefore reported as 0.05 µg/mL. Samples containing bivalent and monovalent natalizumab in known proportions were generated by *in vitro* FAE as described before (22, 30), using a three- to tenfold excess of irrelevant IgG4 (recombinantly produced adalimumab IgG4) (31). Natalizumab F(ab)2 and Fab were prepared as described before (30). Inter-assay variation was 13%. For development and validation of this assay, serum samples for another study stored at Sanquin Laboratory, additional to all serum sample of this research, were used (32). Total natalizumab levels in serum were measured with an ELISA using polyclonal rabbit anti-natalizumab fragments and mouse anti-IgG4 monoclonal antibodies, as previously reported (30). This assay was set up such that spiked natalizumab resulted in similar concentrations with or without FAE. Endogenous IgG4 in serum was measured with ELISA using antihuman IgG4 as capture and antihuman IgG for detection, as described before (33). This assay measures total IgG4 in serum and was used as a proxy for endogenous IgG4, as natalizumab, also an IgG4, is usually present at much lower levels than the endogenous IgG4.

### 2.2 PML cases and controls

Since the initiation of natalizumab treatment for MS patients at the MS Center Amsterdam, located within Amsterdam University Medical Center in the Netherlands, seven natalizumab-treated MS patients were diagnosed with PML. Two patients were diagnosed with carry-over PML, defined as PML diagnosed after switching from natalizumab to ocrelizumab, as described previously (34). All cases are included in the Amsterdam MS cohort (AMSC) and the MS Biobank (Amsterdam UMC Ethics committee numbers 2020.269 and 2016.554, respectively). Each PML case was matched to two control patients selected from the AMSC database. For every PML case, three serum samples were retrieved from the MS Biobank: one collected two years before PML diagnosis, one collected six months before diagnosis, and one collected just prior to diagnosis while still on natalizumab treatment. Matching of PML cases to controls was based on the following criteria using the sample closest to PML diagnosis: age (± 10 years), natalizumab treatment duration (short <2 years or long ≥2 years), natalizumab trough level (± 6 µg/mL), and JCV index value (low <1.5 or high ≥1.5). For each control, one serum sample was obtained from the MS Biobank, selected to match the corresponding PML case as closely as possible based on these criteria. In all retrieved samples, taken at trough, the levels of total natalizumab, bivalent natalizumab and endogenous IgG4 were measured.

### 2.3 Extended interval dosing

To evaluate how FAE is affected by extending natalizumab treatment intervals, we measured total natalizumab, bivalent natalizumab and endogenous IgG4 levels during both standard interval dosing (SID) and EID in stored samples of participants of the NEXT-MS trial. The NEXT-MS trial was a multicenter phase IV non-randomized trial evaluating safety and efficacy of EID of natalizumab in patients with MS (17). Participants could choose to participate in one of the three study groups: the high EID group, aiming for trough levels of 10 µg/mL, the low EID group, aiming for trough levels of 5 µg/mL, or the control group, continuing the standard interval regimen of every four weeks. In total, the NEXT-MS trial included 376 MS patients. Since we wanted to collect one sample during SID with a high trough level (>10 µg/mL), which was measured at baseline of the NEXT-MS trial, and one sample during EID with a low trough level (<10 µg/mL) of each individual, only participants of the EID low group were included in this analysis. The selected samples, all taken at trough, were retrieved form the NEXT-MS biobank and analyzed using the assays described above.

### 2.4 Wearing-off symptoms

To investigate whether FAE plays a role in WoS, we measured the concentrations of total natalizumab, bivalent natalizumab and endogenous IgG4 in participants of the SUPERNEXT trial. This trial, which is an extension of the NEXT-MS trial, is an ongoing phase IV multicenter non-randomized study in which participants receive EID of natalizumab with a minimum interval of six weeks, guided on individual natalizumab trough levels (trial registration number NCT04225312). SUPERNEXT participants complete a WoS questionnaire at baseline, year one, and year two. This questionnaire evaluates whether participants experience WoS during their current treatment, including the specific symptoms and the duration of symptoms, if applicable. This questionnaire has been used previously in other studies (7, 10). For this research, we randomly selected 50 participants reporting WoS and 50 reporting no WoS from all completed questionnaires at baseline. For each participant, the serum sample taken at baseline of the SUPERNEXT study was retrieved from the SUPERNEXT biobank and analyzed using the assays described above. All samples were taken at trough.

### 2.5 Statistical analyses

Expected levels of bivalent natalizumab were calculated by (measured total natalizumab)²/measured endogenous IgG4, assuming proportional distribution of half-molecules of both natalizumab and endogenous IgG4 (35). FAE is the expected dynamic equilibrium reached after several half-lives of the exchange reaction, i.e. roughly 3–7 days after infusion (21, 22). Measured and expected bivalent natalizumab levels were compared using linear regression; samples with measured and/or calculated values below the quantification limit of 0.1 µg/mL were excluded. Additionally, associations between bivalent natalizumab, endogenous IgG4, and total natalizumab were examined by linear regression, using the endogenous IgG4/total natalizumab as predictor and the proportion of bivalent natalizumab (relative to total natalizumab) as outcome.

The association between bivalent natalizumab levels and PML was evaluated using Firth-penalized logistic regression with absolute bivalent natalizumab levels as predictor and PML as binomial outcome, accounting for matched case-control sets (1:2). For PML patients, only the bivalent natalizumab levels measured in the serum samples prior to PML diagnosis were used. Results were considered exploratory due to the limited number of cases. As a sensitivity analysis, the Firth-penalized logistic regression was repeated after exclusion of the two carry-over PML cases.

Individual changes in total and bivalent natalizumab levels between SID and EID were analyzed descriptively and with the Wilcoxon signed-rank test. Associations between bivalent natalizumab levels and the occurrence of WoS were examined using logistic regression with bivalent natalizumab levels as predictor and WoS as binomial outcome, adjusting for sex, age, treatment duration, and the time between questionnaire completion and serum sampling. Additional linear regressions were conducted to explore differences in total natalizumab and endogenous IgG4 levels between patients with and without WoS. Sensitivity analyses excluding samples with bivalent natalizumab values below the quantification limit were performed for EID and WoS analyses. Analyses were conducted using RStudio version 4.2.1, with p-values below 0.05 considered statistically significant. As each hypothesis was evaluated using a distinct dataset, no multiple testing correction was applied.

## 3 Results

### 3.1 Bivalent natalizumab assay

**Figure 2:**
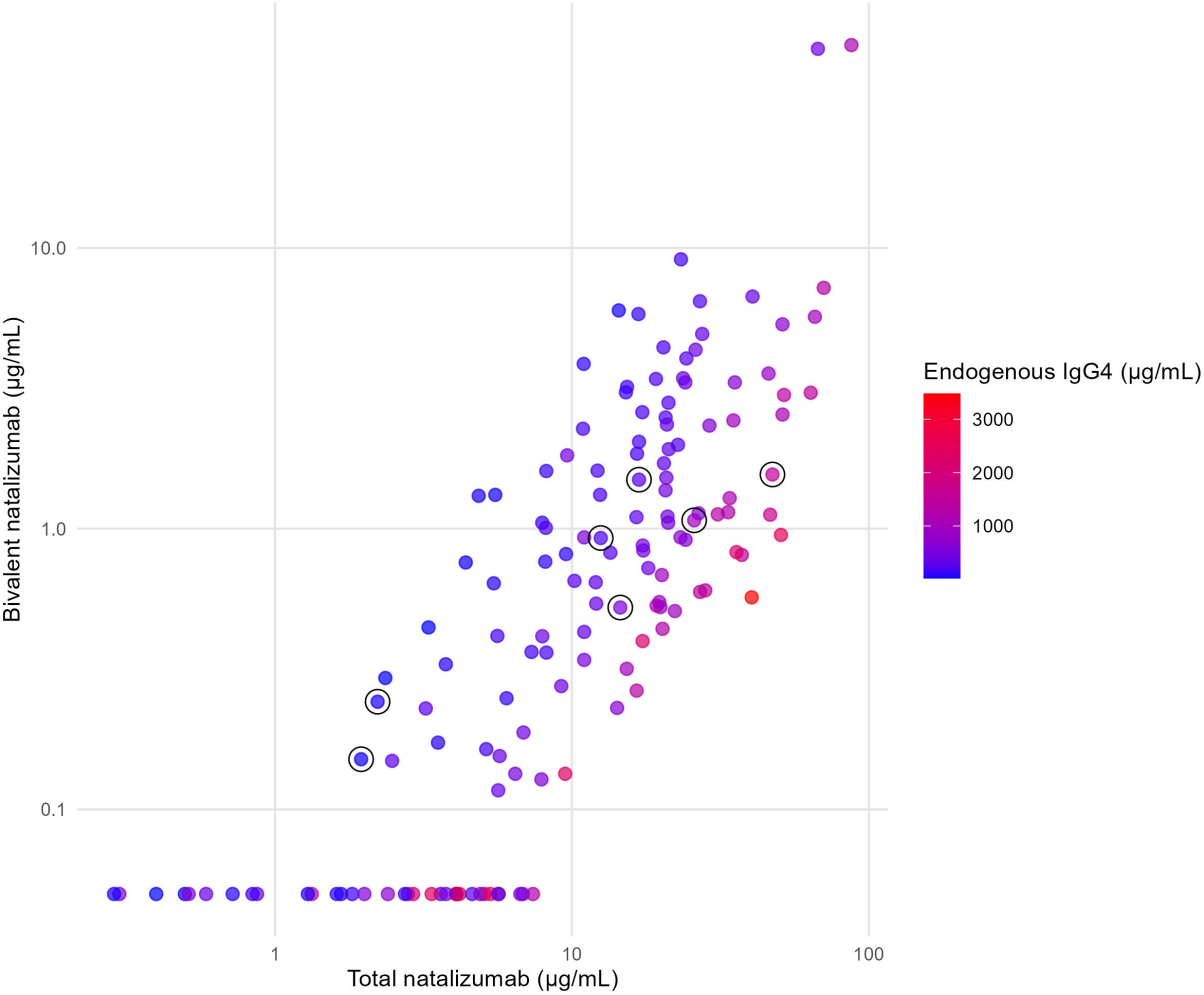
Bivalent natalizumab, total natalizumab and endogenous IgG4 concentrations. Measured bivalent natalizumab versus total natalizumab concentrations in 148 serum samples, both presented on a log scale. Bivalent natalizumab levels below the ELISA quantification limit of 0.10 µg/mL are plotted at 0.05 µg/mL. Color of the dots represent the endogenous IgG4 levels, and circled dots are samples from PML cases that were obtained just before PML diagnosis.

Using 148 serum samples from 103 unique natalizumab-treated patients with MS, an ELISA was set up that specifically only measures bivalent natalizumab (**Supplementary Figure 1**). Specificity was demonstrated by comparing F(ab)2 and Fab fragments of natalizumab; the latter did not render a signal in the assay. Furthermore, upon inducing FAE of natalizumab with an excess of another, irrelevant monoclonal IgG4 antibody, a proportionally lower amount of bivalent natalizumab was measured. Total natalizumab levels in all samples ranged from 0.29 to 87.40 µg/mL, endogenous IgG4 levels ranged from 20.40 to 3480.0 µg/mL and the measured bivalent natalizumab levels ranged from <0.10 to 52.80 µg/mL. The relationship between total natalizumab, endogenous IgG4, and bivalent natalizumab levels is presented in **Figure 2**. It can be appreciated that a higher ratio of endogenous IgG4 to total natalizumab results in lower bivalent natalizumab levels. This was confirmed by statistical analysis, as the ratio of endogenous IgG4 to total natalizumab in serum (median 0.03, IQR 0.02-0.05) was positively associated with the percentage of bivalent natalizumab (β 0.38, 95% confidence interval (CI) 0.27-0.48, p<0.0001). In fact, upon reaching steady state (~3 to 7 days after infusion), one expects the amounts of bivalent and monovalent natalizumab to reflect the relative proportions of total natalizumab and endogenous IgG4 according to simple distribution laws (35). Indeed, we observed that the measured bivalent natalizumab levels closely corresponded to the calculated values (**Supplementary Figure 2**), which was confirmed with analysis in 109 of these samples with bivalent natalizumab levels above the quantification limit of 0.1 µg/mL (log-log regression slope 1.10, 95% CI 1.01-1.18, R²=0.85).

### 3.2 PML cases and controls

For five of the seven PML cases, exact JCV index values were unavailable, though all were known to be above 1.5. Exact JCV index values were only known for the two carry-over PML cases, of which one had a high JCV index value of 2.76, while the other had a low index value of 0.38. The six cases with high JCV index values were matched to twelve controls with similarly high values, with a mean index of 3.12 (range 1.76-4.50). The PML case with a low index (0.38) was matched to two controls with index values of 0.61 and 0.59. **Table 1** summarizes the characteristics of the PML cases and their matched controls, while individual characteristics and measurements are presented in **Supplementary Table 1**. Notably, two PML cases had low natalizumab trough levels in all measurements, ranging from 0.40 to 2.22 µg/mL in one patient, and from 1.95 to 5.52 µg/mL in the other, with both also having low bivalent natalizumab levels.

**Table 1:** Characteristics of PML cases and controls.

Characteristic	PML cases (n=7)			Controls (n=14)
Sex, female, n (%)	3 (43)			9 (64)
Age in years, median (IQR)	40.41 (35.86-42.19)			40.75 (33.14-46.93)
Natalizumab treatment duration in years, median (IQR)	4.73 (3.96-6.35)			6.27 (4.39-6.97)
**Serum sample**	**2 years**	**6 months**	**PML** ^a^	NA^b^
Total natalizumab in µg/mL, median (IQR)	12.45 (8.80-23.30)	13.47 (8.53-19.78)	14.55 (7.36-21.33)	18.01 (8.85-37.72)
Endogenous IgG4 in µg/mL, median (IQR)	359.0 (101.8-736.0)	247.0 (117.8-738.5)	224.0 (110.2-757.0)	254.0 (162.0-576.8)
Bivalent natalizumab in µg/mL, median (IQR)	1.14 (0.53-1.32)	0.87 (0.67-1.46)	0.93 (0.38-1.28)	1.82 (1.02-2.95)

a To compare measurements of PML cases with those of the controls, only results from the sample obtained just before PML diagnosis were used.

b Only one serum sample of control cases was used. The sample was selected based on the matching criteria on age, treatment duration, natalizumab concentration and JCV index. IgG4, immunoglobulin G4; IQR, interquartile range; µg/mL, micrograms per milliliter.

Firth-penalized logistic regression suggested that higher bivalent natalizumab levels may be associated with lower odds of PML (OR 0.30, 95% CI 0.0008-0.82, p=0.013). However, no definite conclusions can be drawn, as the wide CI reflects substantial uncertainty. Furthermore, **Figure 2** shows that PML cases, indicated by circles, do not exhibit distinct levels of total natalizumab, endogenous IgG4, or bivalent natalizumab compared to other natalizumab-treated MS patients. Additionally, in the sensitivity analysis excluding the two carry-over PML cases, the Firth-penalized logistic regression failed to converge due to quasi-complete separation. Also, no consistent trend was observed in bivalent natalizumab levels from two years prior to PML diagnosis up to the last sample before diagnosis (**Supplementary Figure 3**).

### 3.3 Extended interval dosing

In total, 65 participants of the NEXT-MS cohort participated in the EID low study group aiming for 5 µg/mL at trough. Thirty-six of these had a baseline dosing regimen of four weeks and trough levels above 10 µg/mL. Of these, 26 were randomly selected for analyses.

During EID, total natalizumab trough levels were lower than during SID (median 5.30 µg/mL, IQR 4.08-6.80, versus 20.95 µg/mL, IQR 17.28-27.97; Wilcoxon signed-rank test p<0.001). Bivalent natalizumab levels were also lower during EID (median 0.05 µg/mL, IQR 0.05-0.23 versus 2.02 µg/mL, IQR 0.92-3.53; p<0.001). The median individual decrease in total natalizumab between SID and EID was 78% (IQR 67-86), whereas the median individual decrease in bivalent natalizumab was 94% (IQR 87-98). As expected, endogenous IgG4 levels remained relatively stable, with median concentrations of 302.5 µg/mL (IQR 182.5-780.8) during SID and 236.5 µg/mL (IQR 107.5-623.5) during EID. During EID, endogenous IgG4 levels ranged from 20.4 to 2800 µg/mL, and bivalent natalizumab levels ranged from <0.05 to 0.76 µg/mL.

Of the 26 patients, 14 (54%, 95% CI 33-73%) had bivalent levels below the quantification limit (<0.1 µg/mL) during EID, which were imputed as 0.05 µg/mL, whereas none of the SID samples were below the limit (0%, 95% CI 0-13%). When excluding these patients, the remaining 12 had median total natalizumab levels of 17.70 µg/mL (IQR 16.71-21.21) during SID and 6.24 µg/mL (IQR 5.38-7.96) during EID (p=0.003), corresponding to a median individual decrease of 66% (IQR 64-76). Median bivalent natalizumab levels were 2.24 µg/mL (IQR 1.51-3.46) during SID and 0.27 µg/mL (IQR 0.13-0.38) during EID (p=0.003), corresponding to a median individual decrease of 86% (IQR 81-92).

Detailed results for all measurements are presented in **Supplementary Table 2**, and individual changes in bivalent and total natalizumab between SID and EID are shown in **Supplementary Figure 4**.

### 3.4 Wearing-off symptoms

At the time of data extraction, 271 SUPERNEXT participants had completed the baseline WoS questionnaire. Of these, 128 (47%) reported experiencing WoS during natalizumab treatment, while 143 (53%) reported no WoS. From each group, 50 patients were randomly selected. One patient from the group without WoS was excluded due to missing serum measurements. Although baseline WoS questionnaires in the SUPERNEXT study were made available digitally at the time the baseline serum sample was taken, many patients completed the questionnaire after a substantial delay (median 59 days, IQR 12.50-115.0). Analyses were therefore adjusted for this time interval. The median natalizumab dosing interval was 6 weeks (IQR 6-7) in both groups. Detailed baseline characteristics and measurement results are summarized in **Supplementary Table 3**.

**Figure 3:**
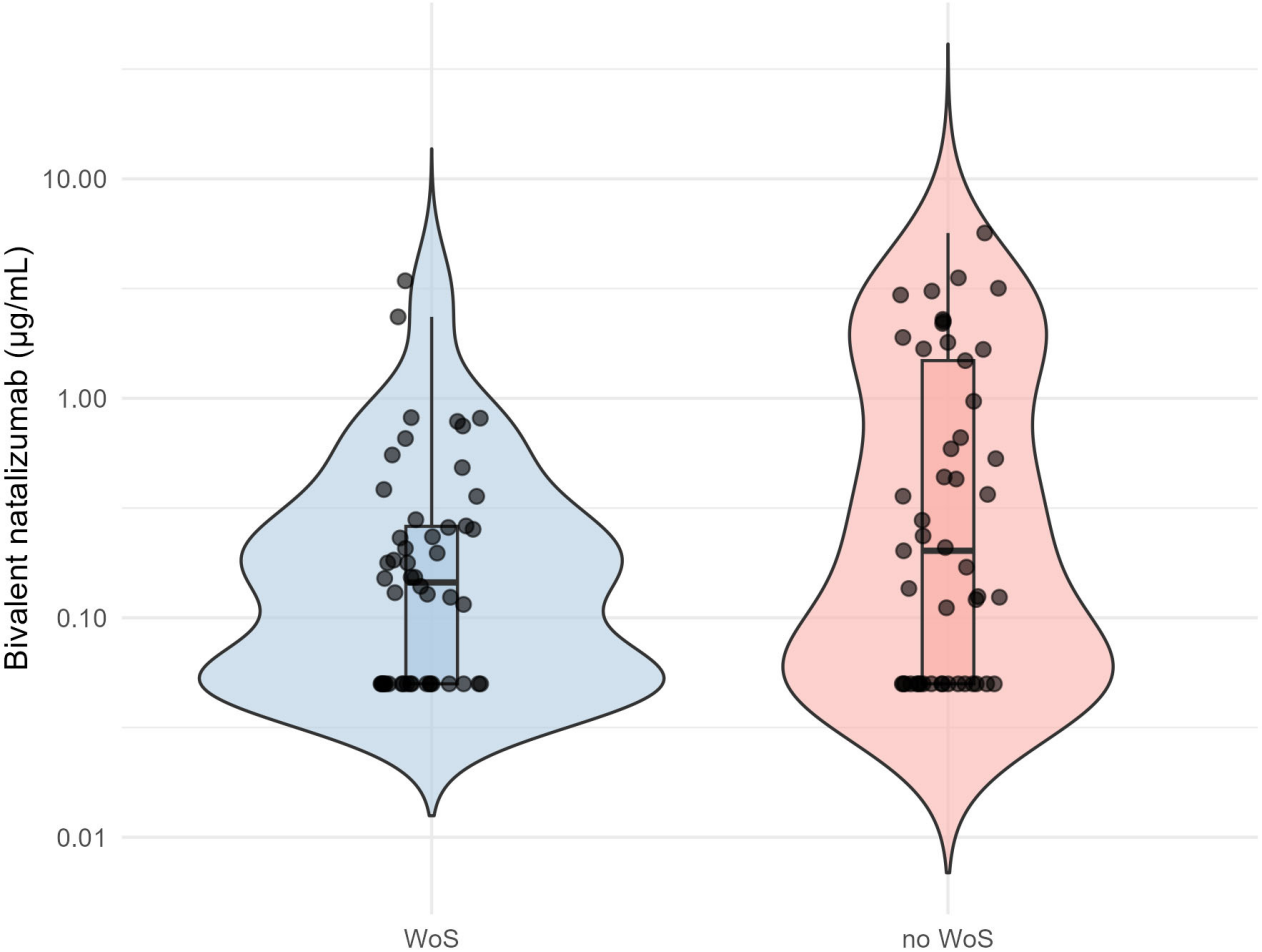
Violin plots of bivalent natalizumab concentrations in patients with and without wearing-off symptoms. Measured bivalent natalizumab, presented on a log scale, plotted for the group of patients with wearing-off symptoms and without wearing-off symptoms. Each dot represents one measurement in an individual. WoS, wearing-off symptoms.

Median bivalent natalizumab levels were lower in the group of patients with WoS (0.15 µg/mL, IQR 0.05-0.26, range 0.05-3.43), compared to those without WoS (0.20 µg/mL, IQR 0.05-1.49, range 0.05-5.67), also when adjusting for sex, age, treatment duration, and the time between questionnaire completion and serum sampling (OR 0.38, 95% CI 0.18-0.68, p=0.004; **Figure 3**). This reflects that patients with WoS had higher endogenous IgG4 levels (median 456.50 µg/mL, IQR 194.80-873.20) than those without WoS (median 243.0 µg/mL, IQR 119.0-510.0) (β 270.56, 95% CI 51.93-489.19, p=0.016), while total natalizumab levels were comparable between the groups: median 7.60 µg/mL (IQR 5.63-12.0) in patients with WoS and median 9.30 µg/mL (IQR 4.60-13.0) in patients without WoS (β -1.86, 95% CI -5.56-1.84, p=0.32).

The number of patients with bivalent natalizumab levels below the quantification limit of 0.1 µg/mL were similar across both groups: 20 (40%) in the group of patients with WoS, and 18 (37%) in the group of patients without WoS. After excluding these patients and repeating the adjusted analysis, median bivalent natalizumab levels remained lower in patients with WoS, with higher levels being associated with a lower odds of WoS (OR 0.33, 95% CI 0.13-0.64, p=0.005).

## 4 Discussion

In this study, we investigated quantitative aspects of FAE, in particular, the relationship between endogenous IgG4 levels and the proportion of circulating bivalent natalizumab. We examined three potential clinical implications of natalizumab FAE by quantifying bivalent natalizumab levels in multiple cohorts of MS patients using a newly developed ELISA. First, no evidence was found to support our hypothesis that bivalent natalizumab levels were higher in patients who developed PML. Second, in patients whose dosing intervals were extended, a proportionally larger decrease in bivalent natalizumab compared to total natalizumab was observed, according to prediction. Third, lower bivalent natalizumab levels were observed in patients who experience WoS.

Natalizumab serum levels are associated with treatment efficacy in natalizumab-treated patients with MS, and can be used to guide treatment intervals in patients receiving EID (17, 36). Although natalizumab levels correlate with receptor occupancy on lymphocytes, studies have shown that receptor occupancy can still vary considerably between individuals with similar natalizumab trough levels (27). A possible reason for this might be the varying endogenous IgG4 levels between individuals, which lead to different bivalent and monovalent natalizumab levels. In the general population, endogenous IgG4 levels range from <10 µg/mL to 2450 µg/mL, with higher levels typically observed in individuals with aeroallergies (37). Consequently, among patients with similar natalizumab trough levels, more FAE is expected in those with higher endogenous IgG4 levels, resulting in a higher proportion of monovalent natalizumab and a lower proportion of bivalent natalizumab. This would likely lead to reduced receptor occupancy, due to the lower receptor avidity of monovalent natalizumab (23–25). However, the specific impact of the ratio between bivalent and monovalent natalizumab on receptor occupancy has not been investigated yet. In this context, it may also be relevant to assess whether bivalent natalizumab levels affect α4β1 integrin expression on lymphocytes, as natalizumab treatment is known to reduce α4β1 integrin expression (38).

The reduced risk of PML found in patients receiving EID of natalizumab is thought to result from the lower trough levels achieved by EID (18). However, PML can still occur in patients with low trough levels, as we also observed in two of the PML cases in our study. Consequently, studies have speculated that bivalent and monovalent natalizumab levels may play a role (21, 25, 26, 28). It was suggested that JCV-positive patients with low natalizumab trough levels could still have an increased PML risk if bivalent natalizumab levels are high, possibly fully restricting lymphocyte trafficking and thereby CNS immune surveillance. The results of this study did not support this hypothesis; we even observed lower bivalent natalizumab levels in PML cases compared to controls, although this analysis should be considered exploratory due to the small number of cases. Further investigation in larger cohorts are required to clarify the potential role of bivalent natalizumab levels in PML risk.

In our study, we confirmed that extending the dosing interval decreases natalizumab trough levels, and, consequently, bivalent natalizumab levels. Notably, the median individual decrease in bivalent natalizumab was greater than that in total natalizumab (94% versus 78%), and remained more pronounced after excluding values below the quantification limit (86% versus 66%). This can be explained by the reduced ratio of total natalizumab to endogenous IgG4, caused by the decrease in total natalizumab, which promotes FAE and leads to a relatively larger reduction in bivalent natalizumab. Furthermore, it was observed that, despite measuring low trough levels (<10 µg/mL) during EID in all patients, bivalent natalizumab levels still varied widely (<0.1-0.76 µg/mL), due to the broad range of endogenous IgG4 levels between individuals (20.4-2800 µg/mL). Therefore, measuring both natalizumab forms, or estimate these based on total natalizumab levels and endogenous IgG4, may allow for an even more personalized approach to determining dosing intervals than relying on total natalizumab levels alone.

The amount of patients reporting WoS at baseline of the SUPERNEXT trial (47%) is comparable to other studies (6–10). So far, the underlying mechanism of WoS remains unclear, and no associations have been identified with EDSS progression or MS disease activity (39). No association was found between WoS and either natalizumab levels or receptor occupancy in a cohort of 93 patients with MS (7). In contrast, another study observed lower receptor occupancy in eight natalizumab-treated patients with MS who regularly reported WoS compared to 22 patients who reported never or only occasionally experiencing these symptoms (8). They suggested that lower receptor occupancy would potentially lead to increased cytokine-releasing lymphocytes trafficking into the CNS, which could induce WoS (6, 7). In our study, we aimed to further clarify this by evaluating bivalent natalizumab levels in patients with and without WoS, given the anticipated impact of bivalent natalizumab on receptor occupancy. We found lower bivalent natalizumab levels in patients who reported WoS, although **Figure 3** shows that bivalent natalizumab levels still spanned the full range in both patients with and without WoS. We therefore remain cautious in drawing conclusions from these findings. Larger studies are needed to better define the potential role of bivalent natalizumab levels in the development of WoS.

Strengths of this study include the broad range of potential clinical implications of natalizumab FAE that were evaluated. Limitations are the small number of PML cases and the absence of receptor occupancy measurements.

Based on our findings, we found that a high proportion of people on EID have low levels of bivalent natalizumab. However, we cannot yet confirm clinical implications of bivalent natalizumab levels in people with MS. As the assumption that bivalent natalizumab is more potent than its monovalent form remains speculative, future studies should incorporate clinical and pharmacodynamic endpoints, such as receptor occupancy. Furthermore, studies on a larger scale are needed to determine whether bivalent natalizumab can be used to further personalize treatment to reduce PML risk and to better define the potential role of bivalent natalizumab levels in WoS.

## NEXT-MS study group members

E.P.J. Arnoldus (Department of Neurology, Elisabeth TweeSteden Hospital, Tilburg, The Netherlands), W.H. Bouvy (Department of Neurology, Diakonessenhuis Hospital, Utrecht, The Netherlands), J.J.J. van Eijk (Department of Neurology, Jeroen Bosch Hospital, ‘s Hertogenbosch, The Netherlands), M. Eurelings (Department of Neurology, Spaarne Gasthuis, Haarlem, The Netherlands), J. van Genugten (Department of Neurology, Ziekenhuisgroep Twente Hospital, Hengelo, The Netherlands), O. N. Groeneveld (Department of Neurology, Isala, Meppel, The Netherlands), E. Hoitsma (Department of Neurology, MS Center Alrijne Hospital, Leiden, The Netherlands), E.L.J. Hoogervorst (Department of Neurology, St Antonius Hospital, Utrecht, The Netherlands), B.A. de Jong (Department of Neurology, MS Center Amsterdam, Amsterdam University Medical Centers, Vrije Universiteit Amsterdam, Amsterdam Neuroscience, Amsterdam, The Netherlands), N.F. Kalkers (Department of Neurology, OLVG, Amsterdam, The Netherlands), M.E. Kloosterziel (Department of Neurology, Wilhelmina Hospital, Assen, The Netherlands), J.J. Kragt (Department of Neurology, Reinier de Graaf Hospital, Delft, The Netherlands), J.P. Mostert (Department of Neurology, Rijnstate Hospital, Arnhem, The Netherlands), C.E.P. van Munster (Department of Neurology, Amphia, Breda, The Netherlands), J. Nielsen (Department of Neurology, Ommelander Hospital, Scheemda, The Netherlands), B.W. van Oosten (Department of Neurology, MS Center Amsterdam, Amsterdam University Medical Centers, Vrije Universiteit Amsterdam, Amsterdam Neuroscience, Amsterdam, The Netherland), L.C. van Rooij (Department of Neurology, Maasstad Hospital, Rotterdam, The Netherlands), C.M. Roosendaal (Department of Neurology, Slingeland Hospital, Doetinchem, The Netherlands), E.A.C. Beenakker (Department of Neurology, Medisch Centrum Leeuwarden, Leeuwarden, The Netherlands), E.M.M. Strijbis (Department of Neurology, MS Center Amsterdam, Amsterdam University Medical Centers, Vrije Universiteit Amsterdam, Amsterdam Neuroscience, Amsterdam, The Netherlands), B.M.J. Uitdehaag (Department of Neurology, MS Center Amsterdam, Amsterdam University Medical Centers, Vrije Universiteit Amsterdam, Amsterdam Neuroscience, Amsterdam, The Netherlands), A. Vennegoor (Department of Neurology, Flevoziekenhuis, Almere The Netherlands), B.H.A. Wokke (Department of Neurology, ErasMS, Erasmus Medical Center, Rotterdam, The Netherlands), E.M.P.E. Zeinstra (Department of Neurology, Isala, Meppel, The Netherlands).

## Data Availability

Anonymized data and associated documentation from this study are available on reasonable request to qualified researchers. Requests to access the datasets should be directed to l.m.y.gelissen@amsterdamumc.nl.
