# Supplementary material for: Clinical implications of natalizumab Fab-arm exchange in patients with multiple sclerosis
Source: Front Immunol. 2026 May 8;17:1796273. doi: 10.3389/fimmu.2026.1796273 (PMC13193995; doi:10.3389/fimmu.2026.1796273)
Supplement: Supplementary file 1 [file Image1.pdf]

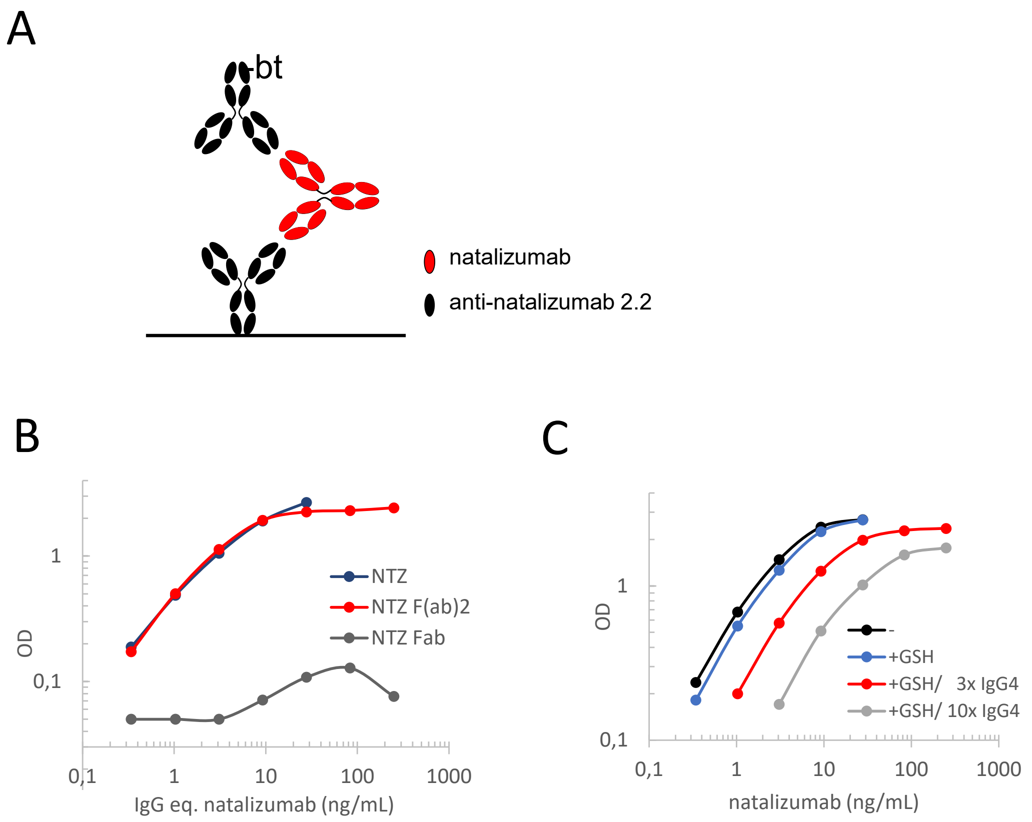


**Supplementary Figure 1.** Assay for measuring bivalent natalizumab.
*A: Assay setup. Natalizumab is captured using a monoclonal anti-natalizumab antibody (clone 2.2), followed by detection using a labeled version of the same antibody. B: The assay only captures bivalent natalizumab, either as intact IgG or as F(ab)2 fragment, but not the monovalent Fab fragment. C: Upon inducing Fab arm exchange with an excess of irrelevant IgG4 antibody, a proportionally lower amount of bivalent natalizumab is detected.*
