# Supplementary material for: Clinical implications of natalizumab Fab-arm exchange in patients with multiple sclerosis
Source: Front Immunol. 2026 May 8;17:1796273. doi: 10.3389/fimmu.2026.1796273 (PMC13193995; doi:10.3389/fimmu.2026.1796273)
Supplement: Supplementary file 2 [file Image2.pdf]

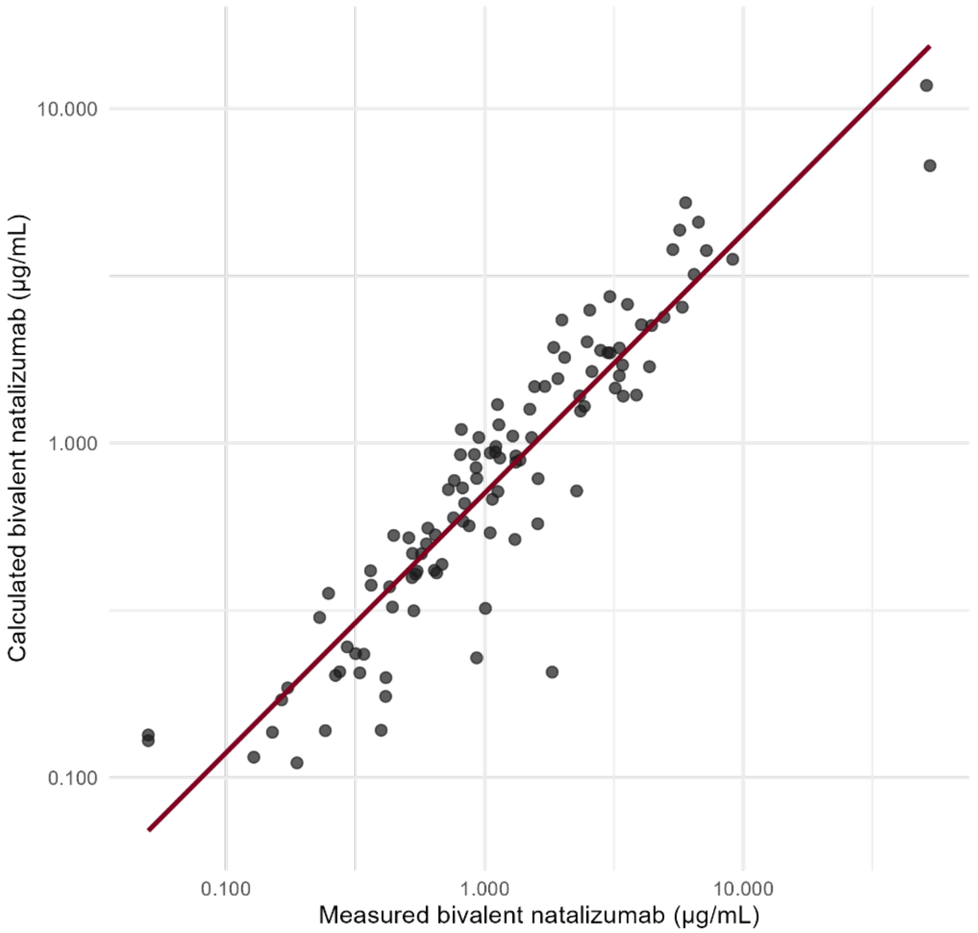

Supplementary Figure 2. Agreement between calculated and measured bivalent natalizumab. *Measured versus calculated bivalent natalizumab levels in 109 serum samples, both presented on a log scale with regression line, with measured and/or calculated bivalent natalizumab levels below the cut-off of 0.1 µg/mL being excluded.*
