# Supplementary material for: Clinical implications of natalizumab Fab-arm exchange in patients with multiple sclerosis
Source: Front Immunol. 2026 May 8;17:1796273. doi: 10.3389/fimmu.2026.1796273 (PMC13193995; doi:10.3389/fimmu.2026.1796273)
Supplement: Supplementary file 3 [file Image3.pdf]

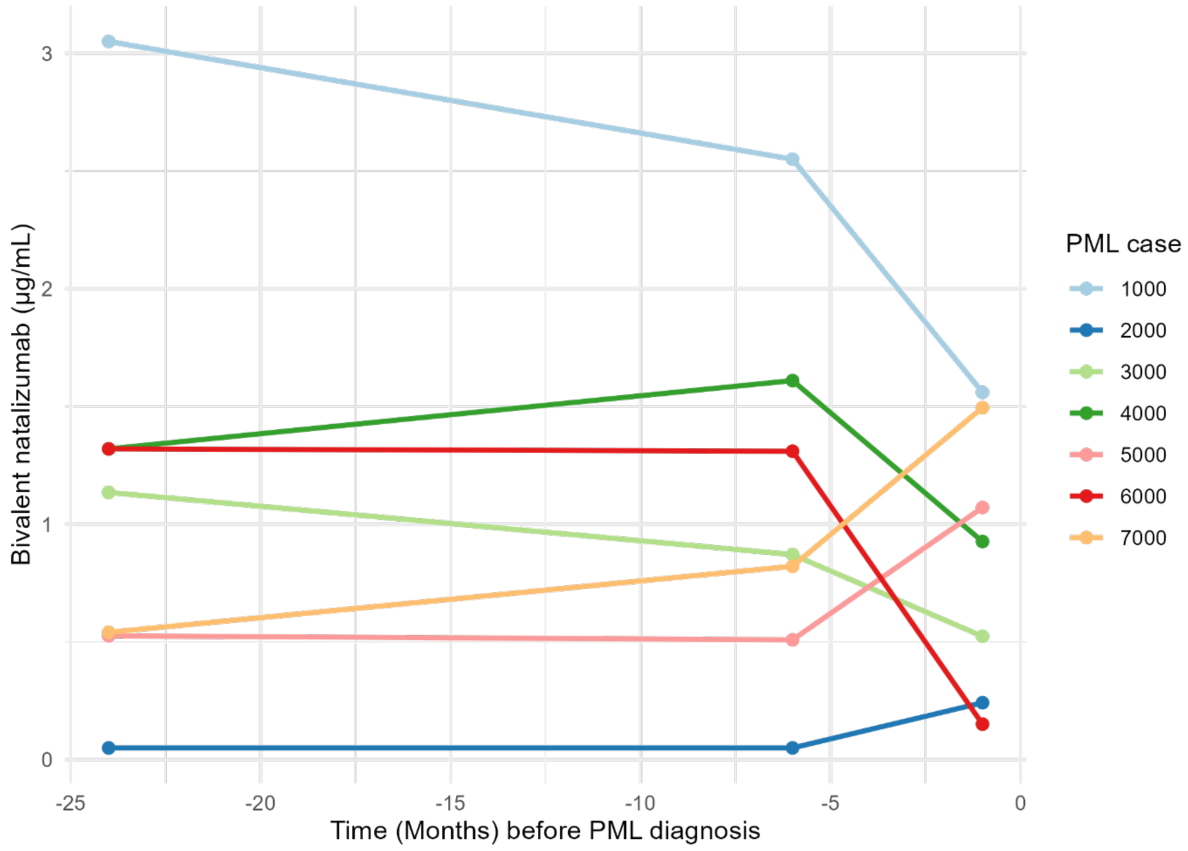

Supplementary Figure 3. Course of bivalent natalizumab in PML cases from two years prior to PML diagnosis.

Each line represents an individual PML case. Natalizumab trough concentrations are shown at three time points: 24 months before PML diagnosis, six months before diagnosis, and immediately prior to diagnosis. Measurements taken after PML diagnosis are not shown.
