# Supplementary material for: Clinical implications of natalizumab Fab-arm exchange in patients with multiple sclerosis
Source: Front Immunol. 2026 May 8;17:1796273. doi: 10.3389/fimmu.2026.1796273 (PMC13193995; doi:10.3389/fimmu.2026.1796273)
Supplement: Supplementary file 4 [file Image4.pdf]

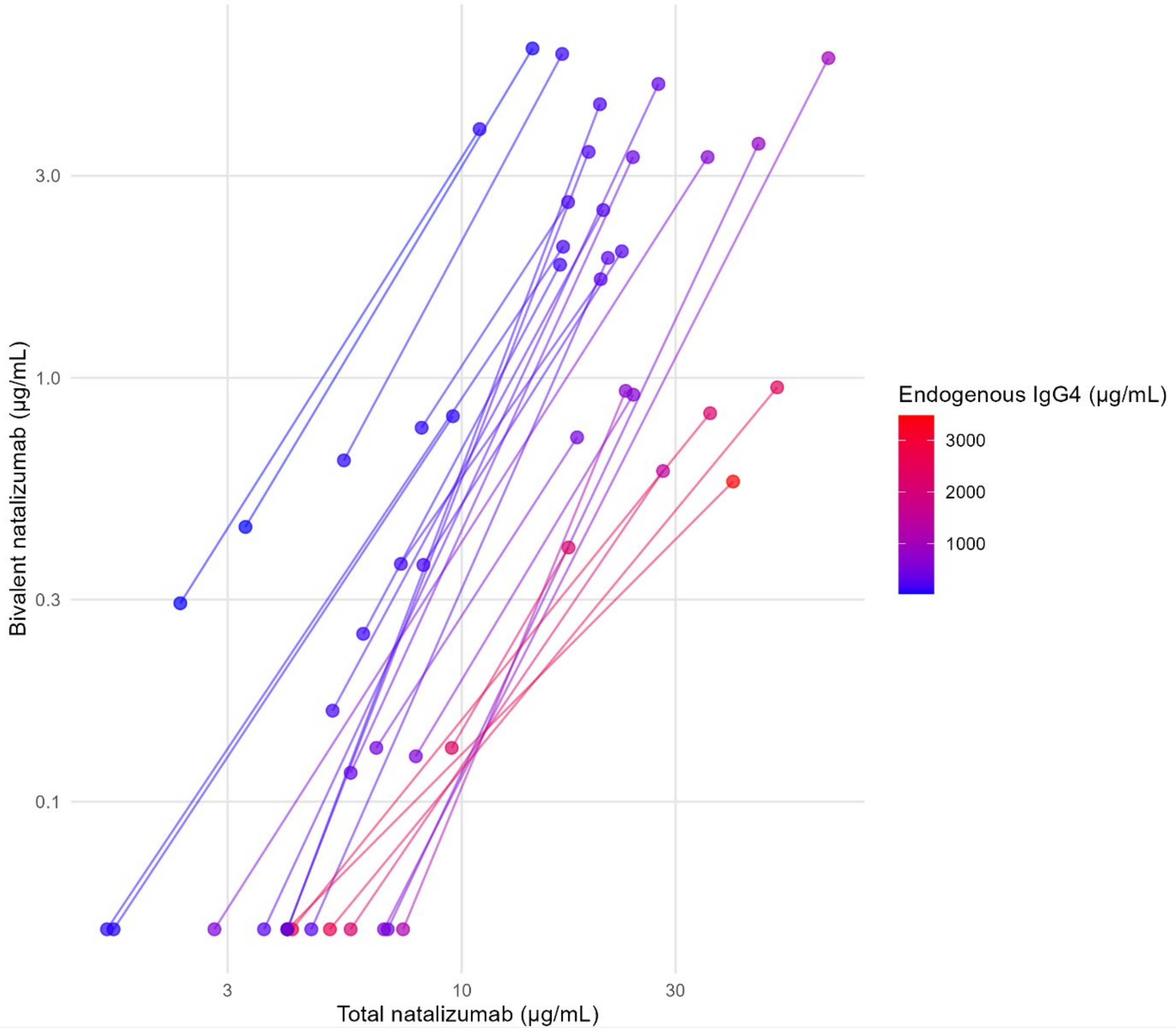

Supplementary Figure 4. Individual bivalent natalizumab, total natalizumab and endogenous IgG4 levels in SID and EID

Upper dots represent individual measurements during SID; lower dots represent measurements during EID. Lines connect paired measurements from the same individual. Bivalent natalizumab levels below the ELISA quantification limit of 0.10 µg/mL are plotted at 0.05 µg/mL.
