# Supplementary material for: Clinical implications of natalizumab Fab-arm exchange in patients with multiple sclerosis
Source: Front Immunol. 2026 May 8;17:1796273. doi: 10.3389/fimmu.2026.1796273 (PMC13193995; doi:10.3389/fimmu.2026.1796273)
Supplement: Supplementary file 5 [file Table1.docx]

**Supplementary Table 1.** Characteristics and measurements of individual PML cases and matched controls

|  | ***Characteristics*** | | | | ***Measurements two years before PML diagnosis*** | | | ***Measurements six months before PML diagnosis*** | | | ***Measurements prior to PML diagnosis / in the matched sample of controls*** | | |
| --- | --- | --- | --- | --- | --- | --- | --- | --- | --- | --- | --- | --- | --- |
| **Subject** | **Sex: male (M) or female (F)** | **Age in years** | **Treatment duration in years^a^** | **JCV index** | **Total NTZ in µg/mL** | **Endogenous IgG4 in µg/mL** | **Bivalent NTZ in µg/mL** | **Total NTZ in µg/mL** | **Endogenous IgG4 in µg/mL** | **Bivalent NTZ in µg/mL** | **Total NTZ in µg/mL** | **Endogenous IgG4 in µg/mL** | **Bivalent NTZ in µg/mL** |
| PML 1 | F | 34 | 4,41 | >1,5 | 63,75 | 1480 | 3,05 | 51,23 | 1050 | 2,55 | 47,38 | 1520 | 1,56 |
| Control 1.1 | F | 43 | 8,07 | 2,02 |  |  |  |  |  |  | 51,20 | 691 | 5,345 |
| Control 1.2 | M | 32 | 12,91 | 4,5 |  |  |  |  |  |  | 40,60 | 360 | 6,715 |
| PML 2 | F | 40 | 1,05 | >1,5 | 0,40 | 21,1 | 0,05 | 0,50 | 23,2 | 0,05 | 2,22 | 35,5 | 0,242 |
| Control 2.1 | M | 29 | 1,01 | 4,38 |  |  |  |  |  |  | 7,95 | 117 | 1,05 |
| Control 2.2 | M | 38 | 4,59 | 3,36 |  |  |  |  |  |  | 3,76 | 68,7 | 0,329 |
| PML 3 | M | 42 | 4,73 | >1,5 | 26,73 | 629 | 1,135 | 17,33 | 530 | 0,871 | 14,56 | 534 | 0,524 |
| Control 3.1 | F | 39 | 4,32 | 3,77 |  |  |  |  |  |  | 21,16 | 236 | 2,81 |
| Control 3.2 | M | 44 | 6,86 | 3,52 |  |  |  |  |  |  | 8,17 | 208 | 1,007 |
| PML 4 | M | 43 | 6,04 | >1,5 | 12,45 | 169 | 1,32 | 12,20 | 190 | 1,61 | 12,51 | 185 | 0,9265 |
| Control 4.1 | F | 48 | 6,48 | 2,32 |  |  |  |  |  |  | 10,90 | 165 | 2,27 |
| Control 4.2 | F | 45 | 6,61 | 3,61 |  |  |  |  |  |  | 15,34 | 161 | 3,2 |
| PML 5 | F | 59 | 3,50 | >1,5 | 19,87 | 843 | 0,526 | 22,23 | 947 | 0,509 | 25,82 | 980 | 1,071 |
| Control 5.1 | F | 51 | 4,75 | 1,76 |  |  |  |  |  |  | 20,68 | 480 | 1,37 |
| Control 5.2 | F | 50 | 8,02 | 3,17 |  |  |  |  |  |  | 29,05 | 609 | 2,33 |
| PML 6^b^ | M | 34 | 6,65 | 2,76 | 5,52 | 34,7 | 1,32 | 4,86 | 45,7 | 1,31 | 1,95 | 27,8 | 0,151 |
| Control 6.1 | F | 50 | 7,00 | 1,96 |  |  |  |  |  |  | 3,54 | 67,5 | 0,173 |
| Control 6.2 | M | 32 | 3,77 | 3,04 |  |  |  |  |  |  | 12,03 | 272 | 0,6445 |
| PML 7^c^ | M | 38 | 10,22 | 0,38 | 12,08 | 359 | 0,541 | 13,48 | 247 | 0,8215 | 16,83 | 224 | 1,495 |
| Control 7.1 | F | 27 | 4,24 | 0,61 |  |  |  |  |  |  | 46,54 | 1660 | 1,1215 |
| Control 7.2 | F | 36 | 6,06 | 0,59 |  |  |  |  |  |  | 51,83 | 1440 | 2,995 |

^a^Natalizumab treatment duration until PML diagnosis (for PML cases) or until the selected sample for control patients.

^b,c^These cases were diagnosed with PML during subsequent ocrelizumab treatment and are therefore referred to as ‘carry-over PML cases’, as described elsewhere.(1)

**References:** 1. Toorop AA, van Lierop ZYG, Strijbis EEM, Teunissen CE, Petzold A, Wattjes MP, et al. Mild progressive multifocal leukoencephalopathy after switching from natalizumab to ocrelizumab. Neurol Neuroimmunol Neuroinflamm. 2021;8(1).
