# Supplementary material for: Clinical implications of natalizumab Fab-arm exchange in patients with multiple sclerosis
Source: Front Immunol. 2026 May 8;17:1796273. doi: 10.3389/fimmu.2026.1796273 (PMC13193995; doi:10.3389/fimmu.2026.1796273)
Supplement: Supplementary file 6 [file Table2.docx]

**Supplementary Table 2.** Bivalent natalizumab during SID and EID

| **Total cohort** | **N=26^a^** | |
| --- | --- | --- |
|  | **SID** | **EID** |
| Total natalizumab in µg/mL, median (IQR) | 20.95 (17.28-27.97) | 5.30 (4.08-6.80) |
| Endogenous IgG4 in µg/mL, median (IQR) | 302.5 (182.5-780.8) | 236.5 (107.5-623.5) |
| Bivalent natalizumab in µg/mL, median (IQR) | 2.02 (0.92-3.53) | 0.05 (0.05-0.23) |
| Percentage bivalent natalizumab of total natalizumab concentration, median (IQR) | 8.90 (4.00-14.75) | 1.70 (1.20-3.90) |
| Ratio endogenous IgG4 : total natalizumab, median (IQR) | 0.07 (0.04-0.11) | 0.02 (0.01-0.05) |
| **Subset** | **n=12^b^** | |
| Total natalizumab in µg/mL, median (IQR) | 17.70 (16.71-21.21) | 6.24 (5.38-7.96) |
| Endogenous IgG4 in µg/mL, median (IQR) | 217.5 (134.0-386.2) | 148.5 (81.97-378.8) |
| Bivalent natalizumab in µg/mL, median (IQR) | 2.24 (1.51-3.46) | 0.27 (0.13-0.38) |

^a^Includes 14 patients with bivalent natalizumab levels below the assay’s quantification limit (<0.1 µg/mL).

^b^Includes only patients with bivalent natalizumab levels during EID above the assay’s quantification limit.

Abbreviations: EID, extended interval dosing; IgG4, immunoglobulin G4; IQR, interquartile range; SID, standard interval dosing; µg/mL, micrograms per milliliter
