# Supplementary material for: Clinical implications of natalizumab Fab-arm exchange in patients with multiple sclerosis
Source: Front Immunol. 2026 May 8;17:1796273. doi: 10.3389/fimmu.2026.1796273 (PMC13193995; doi:10.3389/fimmu.2026.1796273)
Supplement: Supplementary file 7 [file Table3.docx]

**Supplementary Table 3.** Characteristics and measurement results of patients with and without wearing-off symptoms

| **Characteristics** | **Wearing-off symptoms: yes (n=50)** | **Wearing-off symptoms: no (n=49)** |
| --- | --- | --- |
| Sex, female, n (%) | 42 (86) | 39 (78) |
| Age in years, median (IQR) | 40.50 (31.34-53.75) | 42.0 (35.0-51.0) |
| NTZ treatment duration in years, median (IQR) | 5.58 (3.58-9.79) | 6.50 (3.33-10.75) |
| Time between completing questionnaire and serum sampling in days, median (IQR) | 56.0 (11.50-77.0) | 74.0 (16.0-119.0) |
| Natalizumab dosing interval in weeks, median (IQR) | 6 (6-7) | 6 (6-7) |
| **Serum measurements – Total cohort** |  |  |
| Bivalent natalizumab in µg/mL, median (IQR) | 0.15 (0.05-0.26) | 0.20 (0.05-1.49) |
| Endogenous IgG4 in µg/mL, median (IQR) | 456.5 (194.80-873.20) | 243.0 (119.0-510.0) |
| Total natalizumab in µg/mL, median (IQR) | 7.60 (5.63-12.0) | 9.30 (4.60-13.0) |
| **Serum measurements – Subset^a^** | **Wearing-off symptoms: yes (n=30)** | **Wearing-off symptoms: no (n=31)** |
| Bivalent natalizumab in µg/mL, median (IQR) | 0.24 (0.16-0.54) | 0.59 (0.22-2.04) |
| Endogenous IgG4 in µg/mL, median (IQR) | 349.5 (119.8-685.0) | 212.0 (111.0-433.5) |
| Total natalizumab in µg/mL, median (IQR) | 11.50 (7.45-13.0) | 11.0 (9.35-17.50) |

^a^This subset includes only patients with bivalent natalizumab values during EID above the assay’s quantification limit.

Abbreviations: IgG4, immunoglobulin G4; IQR, interquartile range; µg/mL, micrograms per milliliter
